# Prospective clinical study of retinal microvascular alteration after ICL implantation

**DOI:** 10.3389/fcell.2023.1115822

**Published:** 2023-01-19

**Authors:** Chuhao Tang, Yu Zhang, Tong Sun, Jianyang Xie, Yiyun Liu, Rongjun Liu, Zhengze Sun, Hong Qi

**Affiliations:** ^1^ Department of Ophthalmology, Peking University Third Hospital, Beijing Key Laboratory of Restoration of Damaged Ocular Nerve, Beijing, China; ^2^ Cixi Institute of BioMedical Engineering, Ningbo Institute of Materials Technology and Engineering, Chinese Academy of Sciences, Ningbo, China

**Keywords:** implantable collamer lens implantation surgery, myopia, optical coherence tomography angiography, retinal microvasculature, vessel density, visual acuity

## Abstract

**Purpose:** To evaluate the retinal microvascular alteration after implantable collamer lens (ICL) implantation in moderate to high myopia patients using quantitative optical coherence tomography angiography (OCTA).

**Methods:** This prospective cohort study included 50 eyes of 25 patients with preoperative spherical equivalent ≥ −3.00 D. Patients underwent bilateral ICL implantation at the Department of Ophthalmology, Peking University Third Hospital, from November 2018 to July 2019. OCTA was used to image the superficial and deep retinal capillary plexuses before ICL implantation surgery and at 3 months follow-up.

**Results:** There was no significant difference in the microvascular density within each annular zone and all quadrantal zones of the superficial and deep layers found in myopia patients before and after ICL surgery.

**Conclusion:** Levels of microvascular density in retinal capillary plexuses were stable, as detected by the OCTA, showing the high security of ICL implantation, which would not leave adverse effects on retinal microvasculature in myopia patients.

## 1 Introduction

With the increase in educational pressure and limited time outdoors, myopia has become the most common vision problem (Morgan et al., 2018). Recent epidemiological studies indicated a prevalence of myopia as high as 80%–90% in young adults in East Asia (Foster and Jiang, 2014; Wu et al., 2016). It is estimated that, in 2050, half of the world’s population will be affected with myopia and 10% of people will be at a relevant risk of becoming blind as a result of high myopia (Holden et al., 2016; Hopf and Pfeiffer, 2017). With the elongation of the eyeball that occurs with the progression of myopia, the retinal microvascular decrease was observed in the myopia subjects (Yang et al., 2016; Al-Sheikh et al., 2017; Li et al., 2017).

Since the introduction of the Implantable Collamer Lens (ICL; Staar Surgical, Nidau, Switzerland) in 1993, refractive surgery has entered a new era of myopia treatment (Assetto et al., 1996). Because it significantly increased best-corrected visual acuity (BCVA) while reducing caused higher-order aberrations and improving postoperative contrast sensitivity (Wang and Zhou, 2016), the ICL is most frequently used to correct high and extreme myopia. In addition, the ICL performs superbly in the treatment of low to moderate myopia (Kamiya et al., 2012; Dougherty and Priver, 2017; Kamiya et al., 2018).

However, multiple studies revealed that intraocular refractive surgery, such as cataract surgery, may lead to early retinal ischemia, hypoxia, or even retinal vasculitis (Alnawaiseh et al., 2018; Kim et al., 2018; Pilotto et al., 2019; Liu J et al., 2021). Retinal complications would cause a potential or substantial threat to patients’ vision. So, it is vital to monitor retinal microvascular alteration after intraocular refractive surgery. ICL implantation is also a kind of intraocular refractive surgery. However, it is still unknown whether the ICL implantation surgery will affect the retinal microvasculature of myopic eyes.

Optical coherence tomography angiography (OCTA) is a new, non-invasive imaging technique with wide application potential for retinal vascular disease (de Carlo et al., 2015; Gao et al., 2016; Wylegala, 2018). In 2006, Optical Coherence Angiography was first performed to visualize the vasculature in the human eyes (Makita et al., 2006). OCTA can produce high-resolution, three-dimensional images and measure the microvascular network in different layers of the retina structure without the use of contrast agents (Gao et al., 2016; Zhang Q et al., 2016). This study aimed to use OCTA to uncover potential retinal capillary network alterations induced by ICL implantation surgery.

## 2 Materials and methods

### 2.1 Participants

This study includes a total of 50 eyes from 25 participants with moderate and high myopia. All subjects underwent ICL implantation surgery at the Department of Ophthalmology, Peking University Third Hospital between November 2018 and July 2019. Inclusion criteria: 21–45 years old, binocular myopia, with a spherical equivalent of greater than −3.00 diopters (D), anterior chamber depth (ACD) ≥ 2.8 mm, corneal endothelial cell count (cECC) ≥ 2000 cells/mm2, SE remained unchanged for more than 1 year, unsatisfactory vision with contact lenses or spectacles. All patients included in this study had no history of intraocular surgery and showed no other ocular pathologies (uveitis, glaucoma, cataract, keratoconus, severe dry eye, etc.) or serious systemic diseases (diabetes, uncontrolled hypertension, severe hyperthyroidism, etc.).

The method of this study was approved by the Ethics Committee of Peking University Third Hospital (M2020240). In addition, this study was registered and approved on Clinical Trials.gov (NCT04443231). Each subject was given informed consent after an adequate study explanation.

Before surgery, each subject got a full ocular examination: The ACD (measured from the endothelium to the crystalline lens) was measured using anterior segment Optical Coherence Tomography (Visante-OCT; Carl Zeiss Meditec, Jena, Germany), the horizontal white-to-white (WTW) distance and axial length (AL) were measured by optical biometry (IOL Master 700; Carl Zeiss Meditec, Jena, Germany), cECC was obtained from each eye, using a corneal endothelial microscope (SP-2000; Topcon, Tokyo, Japan). Additionally, each eye was subjected to slit-lamp biomicroscopic examination, corneal topography, and funduscopic examination.

The size of the ICL was calculated with a STAAR sizing formula, based on the result of WTW and ACD. Myopia patients are planned for standard ICL implantation surgeries by the same surgeon (QH) under similar settings. Preoperatively, in all patients, 0.5% levofloxacin eye drops were used 3 days before the operation, four times daily and topical anesthesia (4% lidocaine) was administered 30 min before the operation. Through a 3.0-mm temporal corneal incision, the ICL was slowly inserted into the anterior chamber following the implantation of hyaluronic acid (ViscAid, Beijing, China), under visualization with OPMI Lumera 700 surgical microscope (Carl Zeiss Meditec, Germany), and the Toric ICL implantation surgery was completed with the help of the Callisto Eye System (Carl Zeiss Meditec, Germany). Any remaining viscosurgical device was washed out of the anterior chamber with the balanced salt solution. Antibiotic eye drops, steroidal eye drops, and artificial tear drops were used postoperatively.

Moreover, each patient’s eye was assessed for uncorrected visual acuity (UCVA), BCVA, intraocular pressure (IOP), and manifest refraction before surgery as well as 1 day, 1 week, 1 month, and 3 months afterward. For statistical analysis, the decimal Snellen evaluation of UCVA and BCVA was converted to the logarithm of the minimum angle of resolution (logMAR). With the aid of a non-contact tonometer (CT-80; Topcon, Tokyo, Japan), the IOP was measured. The central vault of the ICL (distance from the posterior surface of the ICL to the crystalline lens) was measured using OCT 3 months after surgery.

### 2.2 OCT angiography

After the ocular examination, AngioVue (Optovue, Fremont, CA, United States), was used to capture the OCTA images in all participants from 8:00 a.m. to 12:00 a.m. The system has an A-scan rate of 70 kHz scans per second, with a light source centered on 840 nm and a bandwidth of 45 nm. The scan area was centered on the fovea with a field of view of 6 mm × 6 mm. The resolution of the exported OCT images was 400 × 400 pixels and images with scan quality ≥6 were included for analysis. Automatic segmentation was performed by the software to generate images of the superficial retinal capillary plexus (SCP), and deep retinal capillary plexus (DCP). The SCP was segmented from 3 μm beneath the inner limiting membrane (ILM) to 15 μm beneath the inner plexiform layer (IPL), representing the outer boundary of the ILM to the outer boundary of the IPL. The DCP was segmented from 15 μm beneath the IPL to 70 μm beneath the IPL, representing the outer boundary of the IPL to the outer boundary of the outer plexiform layer (OPL) (Yang et al., 2016).

**FIGURE 1:**
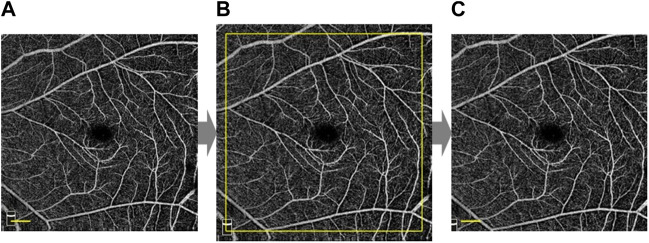
Magnification correction of OCTA high myopia image. According to Bennett’s formula, the original image **(A)** was magnified ×1.10 to obtain the magnified image **(B)** based on AL = 26.17mm, and then further cropped to the size of the original image **(C)**. Scale bar: 600 μm. **(A)**, **(B)**, and **(C)** were based on the same OCTA image.

Due to the elongation of the eye, the magnification for imaging the fundus using fundus photography and OCT differs in the myopic eye (Li et al., 2017). As a result, Bennett’s formula (Figure 1) (Bennett et al., 1994) was used to correct magnification in photographs taken with highly myopic eyes. The correction formula of the image is:

$$\begin{array}{c} t=p\times q\times s \end{array}$$ (1)

Where *t* represents the actual scan length, *p* is the magnification factor determined by the OCTA imaging system, and s represents the original measurement value obtained from the OCTA. The formula of the correction factor *q* is:

$$\begin{array}{c} q=0.01306\times \left(AL-1.82\right) \end{array}$$ (2)

**FIGURE 2:**
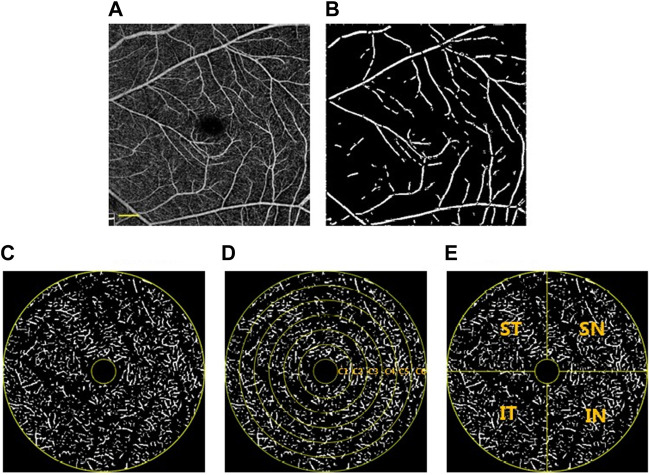
Image partitioning and processing methods. **(A)** OCTA image after magnification correction. The OCTA image was skeletonized and large blood vessels with a diameter> 30 μm were extracted **(B)** and divided. The annular zone with a diameter of 0.6–5 mm **(C)** was divided into 6 annuli (C1—C6) for analysis after the removal of the avascular zone **(D)**. In addition, four quadrants centered on the fovea were generated **(E)**. ST, superior temporal; SN, superior nasal; IN, inferior nasal; IT, inferior temporal. Scale bar: 600 μm. **(A)**, **(B)**, **(C)**, **(D)**, and **(E)** were based on the same OCTA image, which was the same sample as used for Figure 1.

The *AL* is the axial length as mentioned above. The foveal avascular zone (FAZ) centroid was determined using Matlab (The Mathworks, Inc., Natick, MA, United States) and the image was skeletonized. Since large blood vessels in the deep retinal vascular plexus were considered to be projection artifacts of superficial blood vessels (Zhang M et al., 2016), our custom algorithm separated the vessels with diameters >30 μm in both the superficial and deep layers. The images were then converted to binary images. The partition method for the macular retinal region was shown in Figure 2.

Fractal dimension (FD) analysis was commonly used in the objective quantification of retinal capillary complexity (Ab Hamid et al., 2016). Photoshop was used to crop the image of each area separately, and then the box-counting method with FracLab 2.1 toolbox was used to quantitatively analyze the FD (representing blood vessel density) of each area. FracLab (Paris, France) is designed for digital image analysis and is a plug-in for Matlab.

### 2.3 Statistical analysis

The data were presented as the mean ± standard deviation (SD). The differences between the means were evaluated using independent sample t-tests (for patients preoperatively and postoperatively) and analyzed using SPSS Statistics 24 (SPSS Inc., Chicago, IL, United States). *p* < 0.05 was considered significantly different.

## 3 Result

The demographics of the enrolled subjects are summarized in Table 1. The mean patient age at the time of surgery was 27.0 ± 3.8 years (ranging from 21 to 36 years). The preoperative manifest refraction spherical equivalent (MRSE) was −8.50 ± 2.68 D (ranging from −3.50 D to −13.88 D). The preoperative manifest sphere was −7.95 ± −2.57 D (ranging from −3.00 D to −13.75 D). The preoperative manifest refractive cylinder was −1.16 ± 0.92 D (ranging from 0.00 to −3.25 D). The IOP was 14.60 ± 2.71 mmHg. AL was 26.63 ± 1.06 mm (ranging from 24.87 to 29.50 mm). WTW was 11.90 ± 0.31 mm (ranging from 11.3 mm to 12.7 mm). ACD was 3.26 ± 0.25 mm (ranging from 2.92 mm to 3.80 mm).

**TABLE 1:** Preoperative demographics of the myopia patients underwent implantable collamer lens implantation in this study.

Characteristic	Mean ± SD
Number, people/eyes	25/50
Sex, male/female	5/20
Age (years)	27.0 ± 3.8 (range 21–36)
MRSE (D)	−8.50 ± 2.68
LogMAR BCVA	0.02 ± 0.06
AL (mm)	26.63 ± 1.06
WTW (mm)	11.90 ± 0.31
ACD (mm)	3.26 ± 0.25
cECC ( $cells/{mm}^{2}$ )	2913.00 ± 218.86
ICL size (mm)	12.9 ± 0.3
ICL power (D)	−9.38 ± 2.70 (−4.00 to −14.00)

MRSE, manifest refraction spherical equivalent; LogMAR, logarithm of the minimal angle of resolution; BCVA, best corrected visual acuity; AL, axial length; WTW, white-to-white; ACD, anterior chamber depth; cECC, corneal endothelial cell count.

**FIGURE 3:**
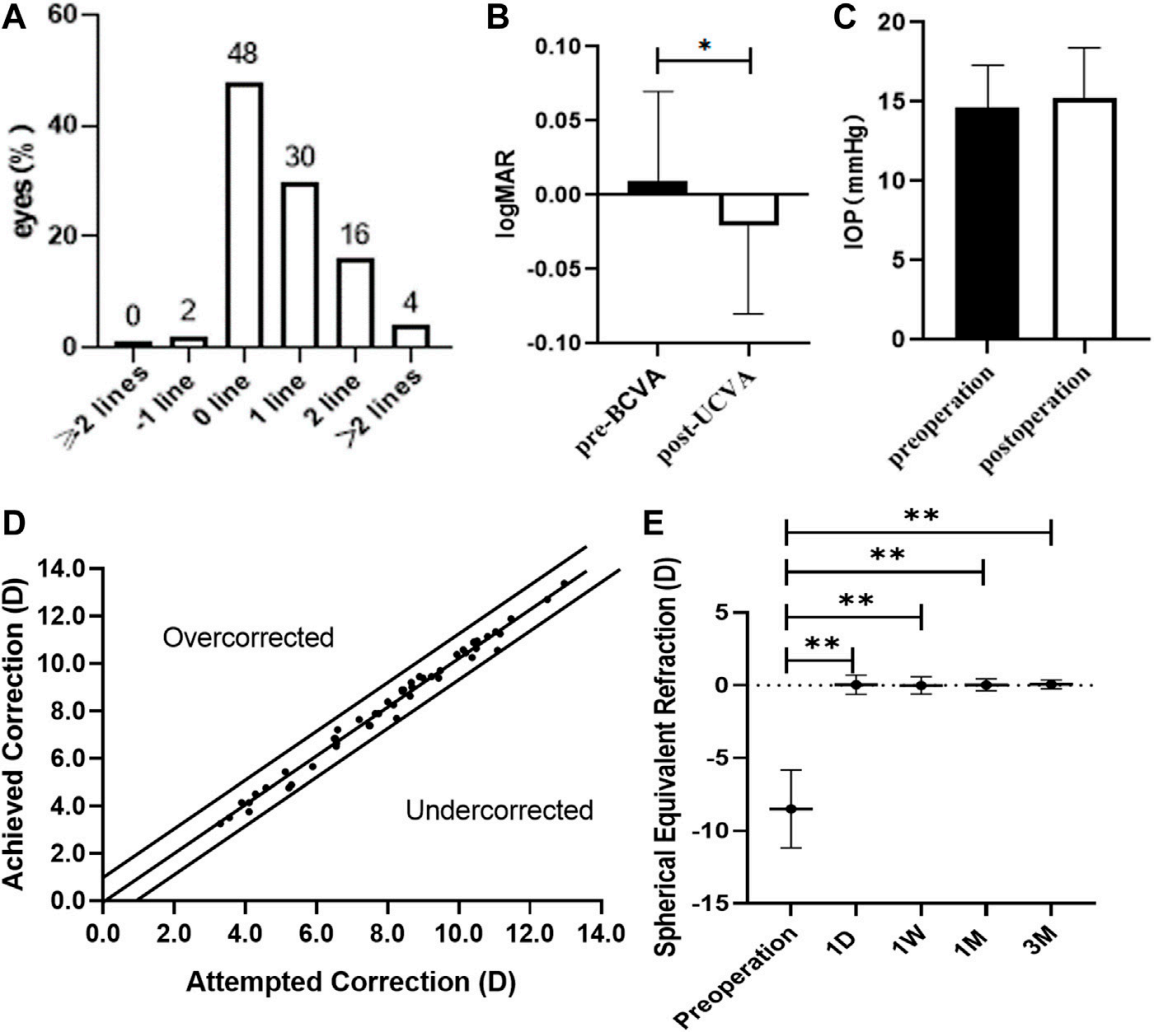
Clinical examinations of myopia patients after ICL implantation surgery. **(A)** Changes in Snellen lines of BCVA at 3 months after ICL implantation. **(B)** Changes between BCVA 3 months after ICL implantation and UCVA Preoperatively. **(C)** Changes in intraocular pressure 3 months after ICL implantation. **(D)** A scatter plot of the attempted versus the achieved manifest spherical equivalent correction 3 months after ICL implantation. **(E)** Time course of manifest spherical equivalent after ICL implantation. The asterisks indicate statistically significant differences between pre-surgery and post-surgery. D, day; W, week; M, month(s).

All surgical procedures were uneventful, and no postoperative complications, such as cataract formation, pigment dispersion syndrome, pupillary block, or axis rotation, were seen throughout the observation period. Visual acuity improved for all patients on the first day after surgery. Three months postoperatively, 2% of eyes lost one line of vision and 98% of eyes maintained or gained BCVA (Figure 3A). The efficacy index was 1.11 ± 0.24 (preoperative BCVA: 0.01 ± 0.06 logMAR and postoperative UCVA: −0.02 ± 0.06 logMAR, *p* < 0.05; Figure 3B). The mean preoperative IOP and postoperative IOP (14.6 ± 2.7 versus 15.2 ± 3.2, *p* > 0.05; Figure 3C) were not significantly different. At 3 months postoperatively, 90% and 100% were within ±0.5 and 1.0 D of the attempted correction, respectively (Figure 3D). The time course changes in the manifest refraction were shown in Figure 3E. Changes in the manifest refraction from 1 day to 3 months were 0.02 ± 0.68 D. Three months postoperatively, the vault was 0.64 ± 0.20 mm.

**FIGURE 4:**
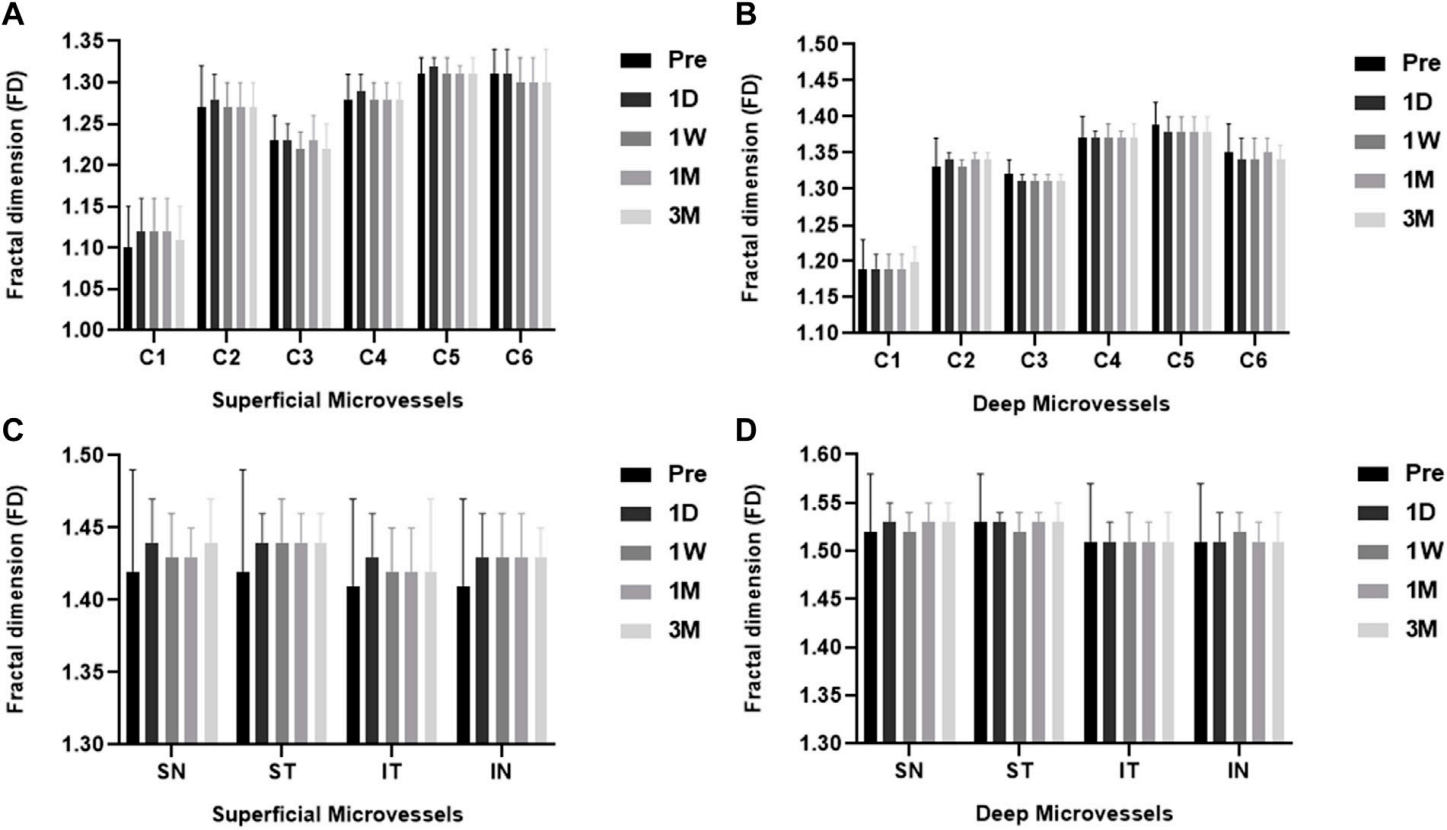
The FD (representing retinal microvascular density) of ICL patients pre- and post-surgery for each layer. Among the six individual annular zones **(A, B)**, the microvascular density in the **(A)** superficial and **(B)** deep layers showed no significant difference in myopia patients pre- and post-surgery (all *p* > 0.05). The microvascular density was also not significantly different in all quadrantal zones of the superficial **(C)** and deep **(D)** layers in myopia patients pre-and post-surgery. (all *p* > 0.05). ST, superior temporal; SN, superior nasal; IN, inferior nasal; IT, inferior temporal.

Preoperative versus postoperative retinal microvascular density for myopia patients, with *p* values for comparison. The total annular zone was divided into four quadrantal zones and six annular zones (bandwidth = 0.73 mm). No significant difference in the microvascular density within each annular zone and all quadrantal zones of the superficial and deep layers was found in myopia patients between pre-surgery and post-surgery (Figure 4).

## 4 Discussion

Myopia is one of the most prevalent eye disorders, and the epidemic of high myopia, in particular, is a serious hazard to public health, such as financial, psychological, quality of life, and direct and indirect risks of blindness. ICL implantation is one of the methods to treat myopia. According to our data, ICL implantation is a safe and effective treatment for both moderate and high myopia. No eye loses two or more lines of vision after ICL implantation. All eyes were within ±1.0 D of the attempted correction and both refractive status and IOP remained stable for 3 months after surgery. Its performance in safety, effectiveness, stability, and predictability is even better than the results of Sanders et al. (2004) due to the advancement of surgical techniques and the update of ICL.

Retinopathy is the most common complication of high myopia, which is a slowly progressive and sight-threatening condition. Several studies have investigated the retinal microvascular in patients with myopia, revealing the retinal microvascular network alterations in myopic eyes. The structural elongation of the eyeball mechanically stretches the retinal tissue, resulting in the straightening and narrowing of the microvessels and consequently the decrease of the retinal microvascular density and perfusion in myopic eyes (Li et al., 2017; Leng et al., 2018; Li et al., 2018; Milani et al., 2018; Gołębiewska et al., 2019; Guo et al., 2019). Jiang et al. (2021) found that the superficial and deep macular microvascular density in high myopia was significantly higher than that in non-high myopia by using OCTA; Liu M et al. (2021) also reached the same conclusion in a larger sample study and found a negative correlation between microvascular density and axial length.

OCT angiography, as an advanced imaging technique characterized by non-invasiveness, quantification, and reliability, which can detect blood flow signals in the retina, has superior advantages over traditional angiography techniques. OCTA is widely used in the evaluation and diagnosis of eye diseases, such as high myopia retinopathy (Grudzińska and Modrzejewska, 2018), glaucoma optic nerve damage (Rao et al., 2020), retinal vein occlusion (Hirano et al., 2021) and diabetic retinopathy (Sun et al., 2021). Using OCTA, images from different layers of the retina can be projected clearly due to its high resolution. In the present study, by using OCT angiography, superficial and deep retinal capillary density were measured in moderate and high myopia patients who underwent ICL implantation between pre-surgery and post-surgery. The OCTA provided the macular perfusion of a 6 mm × 6 mm area, we calculated the microvascular density in each region through the box-counting method after correcting for magnification. Our data showed that ICL implantation surgery would not leave adverse effects on the retinal capillary network.

Moreover, clinical evidence suggests that eye surgery would cause the alteration of retinal microcirculation (Alnawaiseh et al., 2018; Kim et al., 2018; Liu J et al., 2021). Pilotto et al. (2019) found that the perfusion of the retinal microvascular plexus in the deeper layers of the macula increased after uncomplicated cataract surgery, which may be related to the early postoperative local inflammatory response. Analogously, Chen et al. (2017) used a retinal oximeter to detect retinal oxygen desaturation due to retina oxygen deficiency after ICL implantation. ICL implantation surgery as a safe and effective refractive surgery plays an important role in correcting moderate and high myopia. However, due to the unusual anatomy and physiology of the retina in high myopia eyes, it deserves our attention for any microcirculation abnormality inherent to ICL implantation surgery. Using OCTA, ophthalmologists can easily assess patients’ retinal microvascular health and disease, which might be beneficial for pre-operative evaluation of ICL implantation surgery and the detection of postoperative complications.

There were a few limitations to this study. Since the levels of microvascular density in the retinal capillary plexuses after ICL surgery detected by OCTA were stable in this study, the sample size could not be calculated. Although we have included as many participants as possible in this study, the number of patients was relatively small and the follow-up period was only 3 months. In bigger populations and during longer follow-up periods, retinal microvascular change may be seen. Besides, because most of the patients who underwent ICL implantation were young people, this study did not include children and the elderly, whose preoperative retinal microcirculation is slightly different (Leng et al., 2018; Golebiewska et al., 2019), and might respond differently to ICL implantation surgery. Moreover, the OCTA scan area used in this study is 6 mm × 6 mm around the macula. Although it has a wider scan range, it has not achieved the best presentation on retinal microvasculature.

In conclusion, this study demonstrates that ICL implantation is an effective treatment for both moderate and high myopia in young patients, with excellent safety, predictability, and stability. Simultaneously, using OCTA, this research provides evidence that ICL implantation has no adverse effects on retinal microvascular. Further larger sample sizes and longer-term studies are warranted to confirm the conclusions presented herein.

## Data Availability

The raw data supporting the conclusion of this article will be made available by the authors, without undue reservation.
